# Parallel and functionally segregated processing of task phase and conscious content in the prefrontal cortex

**DOI:** 10.1038/s42003-018-0225-1

**Published:** 2018-12-05

**Authors:** Vishal Kapoor, Michel Besserve, Nikos K. Logothetis, Theofanis I. Panagiotaropoulos

**Affiliations:** 1 Department of Physiology of Cognitive Processes, Max Planck Institute for Biological Cybernetics, 72076 Tübingen, Germany; 2 Graduate School of Neural and Behavioral Sciences, International Max Planck Research School, Eberhard-Karls University of Tübingen, 72074 Tübingen, Germany; 3 Department of Empirical Inference, Max Planck Institute for Intelligent Systems and Max Planck ETH Center for Learning Systems, 72076 Tübingen, Germany; 4 Imaging Science and Biomedical Engineering, University of Manchester, Manchester, M13 9PL UK; 5 Cognitive Neuroimaging Unit, CEA, DSV/I2BM, INSERM, Universite Paris-Sud, Universite Paris-Saclay, Neurospin Center, 91191 Gif/Yvette, France

**Keywords:** Visual system, Neural circuits, Consciousness, Sensory processing

## Abstract

The role of lateral prefrontal cortex (LPFC) in mediating conscious perception has been recently questioned due to potential confounds resulting from the parallel operation of task related processes. We have previously demonstrated encoding of contents of visual consciousness in LPFC neurons during a no-report task involving perceptual suppression. Here, we report a separate LPFC population that exhibits task-phase related activity during the same task. The activity profile of these neurons could be captured as canonical response patterns (CRPs), with their peak amplitudes sequentially distributed across different task phases. Perceptually suppressed visual input had a negligible impact on sequential firing and functional connectivity structure. Importantly, task-phase related neurons were functionally segregated from the neuronal population, which encoded conscious perception. These results suggest that neurons exhibiting task-phase related activity operate in the LPFC concurrently with, but segregated from neurons representing conscious content during a no-report task involving perceptual suppression.

## Abstract (web summary)

Vishal Kapoor et al. identify a population of cells in the lateral prefrontal cortex that exhibits task phase-related activity during a no-report task. This cell population is functionally segregated from the population encoding conscious perception, although the two operate in parallel.

## Introduction

A major focus in the pursuit to unravel the neural correlates of consciousness has been on investigating the physiological activity underlying the contents of conscious perception^1–4^. This approach has thus far revealed that the proportion of neurons (as well as their strength of modulation) whose activity correlates with subjective perception increase as one progresses in the visual cortical hierarchy, peaking to ~90% in the primate temporal lobe, compared to ~20% in the primary visual cortex^1,4^. LPFC is the final region of the ventral visual pathway with reciprocal anatomical connections with the temporal lobe^5^ and neurons in this region display responses selective to complex stimuli such as faces and objects^6–10^. In a previous study^11^, we exploited this selectivity for visual features^7,8^ in the LPFC, in order to understand its role in visual awareness. Using a no-report task^12^ of binocular flash suppression (BFS)^13,14^, which allows the successful dissociation of sensory input from phenomenal awareness, it was found that the majority of stimulus selective cells (60–90% depending upon the strength of selectivity) encoded subjective visibility^11^. This finding gave credence to the ‘frontal lobe hypothesis’^15^, and theoretical frameworks such as the higher order^16^ and global workspace theories^17,18^ which postulate PFC as an essential anatomical node mediating consciousness.

However, recently the role of LPFC in mediating conscious perception has been a topic of intense discussion^3,12,19–23^ mostly because of its concomitant role in mediating task relevant behavior and motor action^24^. Indeed, a major role attributed to the LPFC is the temporal organization of behavior^25–27^. Neurons in this region encode aspects relevant to the current task; not just the sensory input as discussed above, but also abstract cognitive processes responsible for successful behavior such as decision making, working memory, reward expectation and temporal sequencing of sensory input or motor action, to mention a few^24,26,27^.

The present study examined the possibility that segregated LPFC populations encode conscious content and task-phase related activity during a no-report paradigm. Such an investigation is both essential and timely, given the recent advocacy towards utilizing no-report tasks for investigating conscious perception because of their presumed ability to circumvent task related activations^12^. Moreover, no-report does not necessarily imply absence of task relevant activity as it is commonly assumed. To this end, we characterized the major CRPs present among LPFC neurons during a no-report task of BFS. Our results show that a large proportion of units were modulated in relation to task phase, and were unaffected by sensory context or perceptually suppressed input. Further, the structure of spike count correlations among neurons clustered to the various CRPs displayed a functional organization akin to orientation selective neurons in primary visual cortex, and these correlations were similar across task conditions^28^. Most importantly, correlation analysis revealed that stimulus selective units were functionally segregated from units, which displayed task-phase related activity. Together, our results suggest that different and functionally segregated neural populations in the LPFC encode conscious perception and task monitoring functions and operate in parallel during a no-report task of perceptual suppression.

## Results

### BFS and neuronal responses in the LPFC

**Fig. 1:**
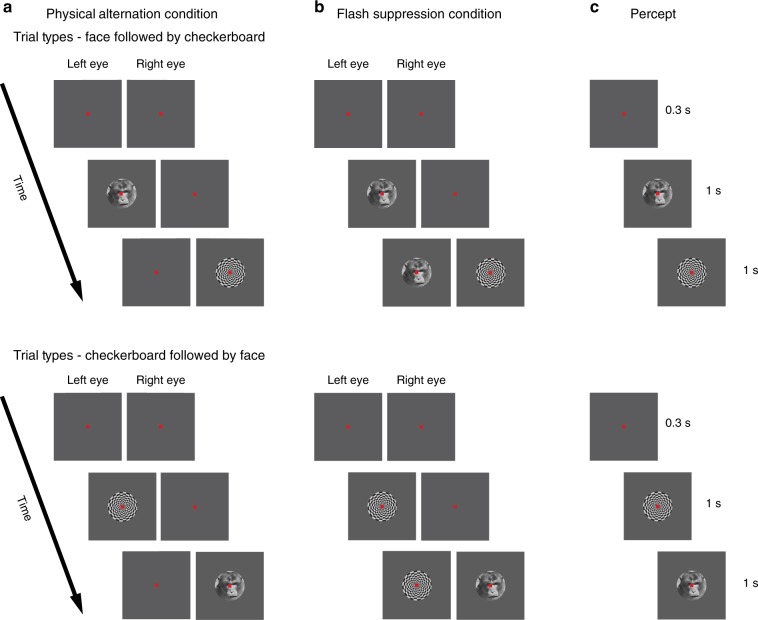
Time line of the binocular flash suppression paradigm. The task consisted of two conditions, namely **a** physical alternation (PA) and **b** flash suppression (FS). The percept is shown in **c**. Each trial started with a fixation spot presented to both eyes for 0.3 s, after which a face or a checkerboard pattern was monocularly presented for one second. Following this, a second stimulus was added to the contralateral eye after extinguishing (in PA) or without the removal of the first stimulus (in FS). The time axis next to each trial type indicates trial chronology, starting at the top, and finishing at the bottom. Stimuli presented during the two conditions in the upper panel elicit a perceptual transition from a face to a checkerboard pattern, while in the lower panel, the order of stimulus presentation is reversed. Note that although the sensory stimulation is different across the two conditions, PA (**a**) and FS (**b**), the subject’s percept (**c**) is the same within the two trial types. Trial types presented in the figure comprise half of the trials, where the first visual pattern was presented in the left eye. In the rest of the trials, the first visual pattern was presented in the right eye (refer to the Methods section for details)

Electrophysiological activity was recorded from 1285 single units with twisted wire tetrodes^29^ in the LPFC of three animals while they passively fixated during the task of BFS (Fig. 1). Units displaying stimulus preference (*N* = 151) have been previously described^11^ and were removed from further analysis (Supplementary Fig. 1). In addition, we also omitted single units with very low spiking activity (*N* = 51) (see Methods section) from further analysis and we report here the results pertaining to the remaining neurons (*N* = 1083).

BFS is a no-report task^12^ that permits the dissociation of sensory input from phenomenal perception^13,14^. The experimental design consists of two different trial types, the physical alternation (PA) (Fig. 1a) (control trials) and flash suppression trials (FS) (Fig. 1b). Each trial type starts with a fixation spot presented binocularly (through a stereoscope) that cues the animal to fixate. Following successful fixation for 300 ms, a stimulus (typically a face or a checkerboard pattern overlaid by the fixation spot as depicted in Fig. 1) was presented monocularly for one second. Following this, in PA trials, the first stimulus was extinguished from the first eye and a second stimulus was presented to the contralateral eye. In FS trials, which were randomly interleaved with PA trials, the second stimulus was added to the contralateral eye without the removal of the first stimulus. This results in the robust perceptual suppression of the first stimulus, which typically lasts longer than one second^11,30^. The presentation of the second stimulus lasted one second and upon successful fixation for the entire duration of the trial, the animals were given a liquid reward. Perception in the two trial types is identical (Fig. 1c), but distinct underlying stimulation in the second half of the trial permits assessing if the neural activity is driven by sensory input or modulated by the phenomenal percept.

**Fig. 2:**
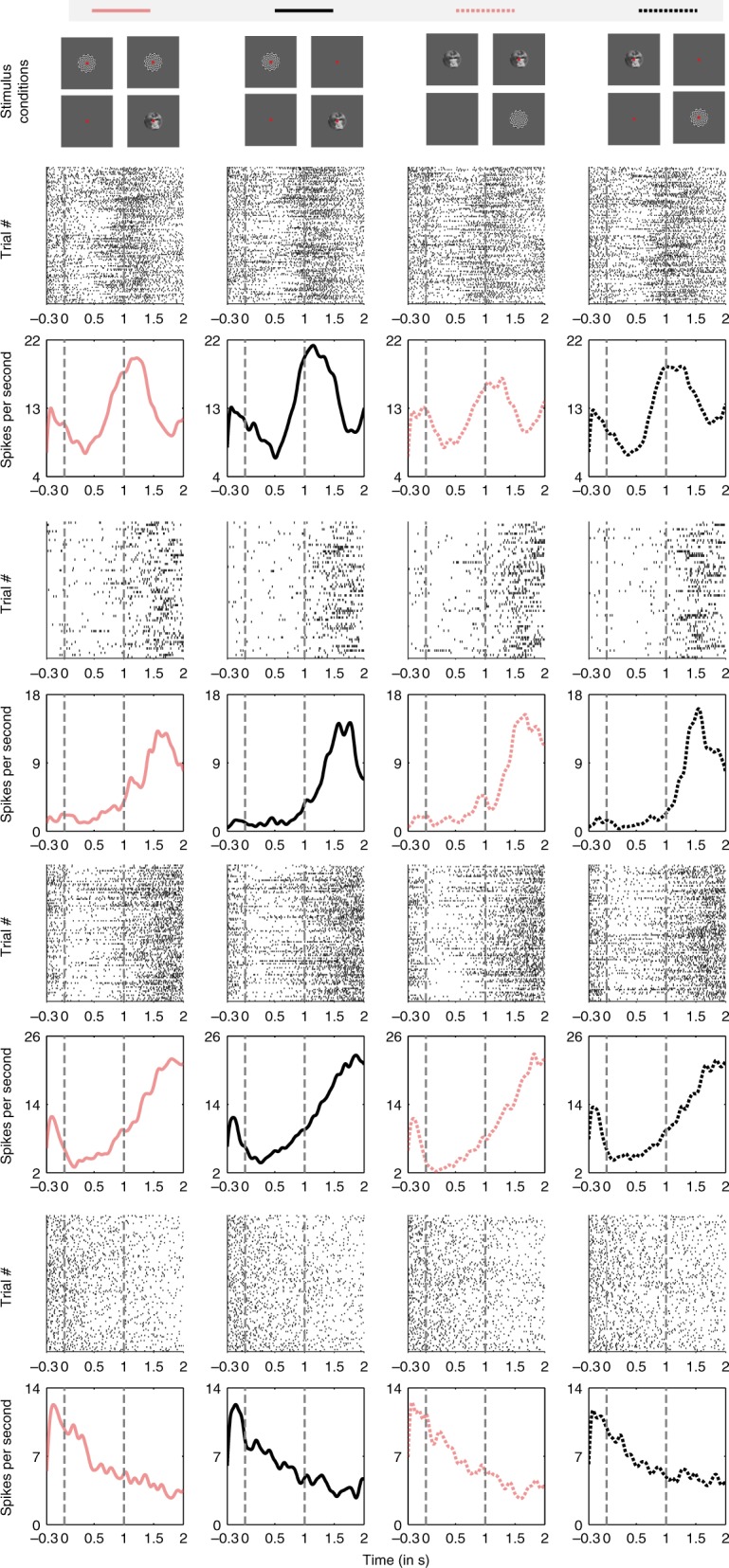
Responses of four example single units during PA (black) and FS (pink). Each neuron’s single trial response (Trial # axis refers to different trials) is shown using raster plots. Below the raster plots is the PSTH of each neuron. Solid line plots indicate the neuronal responses when checkerboard pattern is presented first followed by the face stimulus. Neuronal responses when the face is presented first followed by the checkerboard pattern are plotted with dotted lines. The stimulus conditions are also displayed in the uppermost panel. Some cells tend to fire in an earlier phase of the trial and some later. Across neurons, a considerable heterogeneity in their response patterns can be observed. Further examples are displayed in Supplementary Fig. 2

Figure 2 shows response profiles and raster plots of example neurons recorded during the task (additional examples are presented in Supplementary Fig. 2). An initial visual inspection indicates that neuronal activity displays different patterns. These included units with a ramping up or a ramping down nature of activity as well as neuronal responses limited to the first or the second half of the trial. Three distinct observations can be made from the nature of the response profiles. First, single unit responses in the LPFC are quite heterogeneous. Second, the discharges of these neurons were usually temporally restricted to different and specific phases of the task. Finally, the neuronal response profiles were very similar across the four different stimulus conditions (see the Methods section for a description of the stimulus conditions). The time courses of these neuronal responses are distinct from neurons displaying stimulus selectivity (Supplementary Fig. 1).

### Categorization of neuronal response patterns

**Fig. 3:**
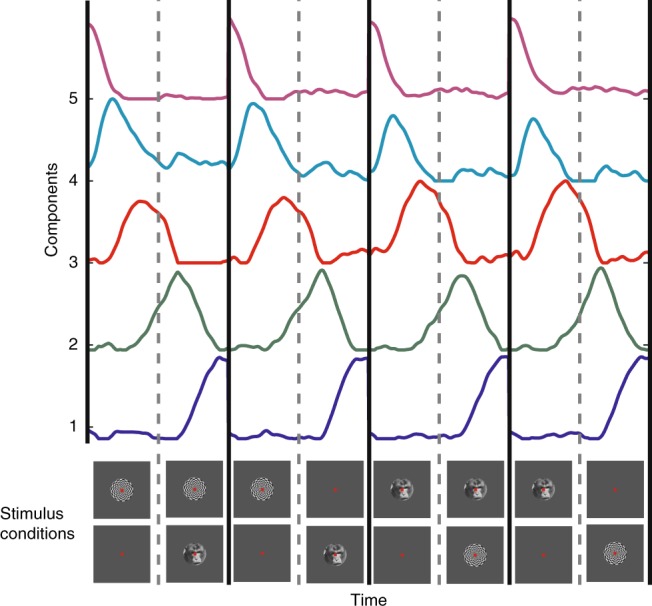
Canonical response patterns (CRPs) obtained by decomposing the single unit responses, with non-negative matrix factorization (NNMF) (refer to text for the details of the clustering procedure). Five CRPs were obtained, with their peak amplitudes distributed through different phases of the task. The lower panel displays the stimulus presentation conditions, demarcated by black lines. See also Supplementary Figs. 3 and 4

In order to identify and differentiate between the diverse patterns of single unit responses, a non-negative matrix factorization (NNMF)^31,32^ based decomposition of response profiles was performed (see Methods for details, and Supplementary Figs. 3 and 4). With this method, we identified five CRPs, which summarized the activity of all recorded single units (Fig. 3). These five CRPs accounted for 87.7% of the energy of the response data matrix. The energy of the residual error of this approximation with NNMF was 13.3% of the energy of the data matrix (see Methods section). Two CRPs exhibited their peaks close to two salient events, namely the beginning or end of the trial. Another two showed peaks in their amplitude after the first or the second presented stimulus. In addition, one CRP displayed a response pattern, which increased its activity toward the expected stimulus switch and then a decrease thereafter. Moreover, the CRPs were virtually identical across the different stimulus conditions, suggesting that the critical factor for these modulations was trial phase. In summary, the patterns were homogenously distributed across the entire trial duration, segmenting it in five distinct phases.

**Fig. 4:**
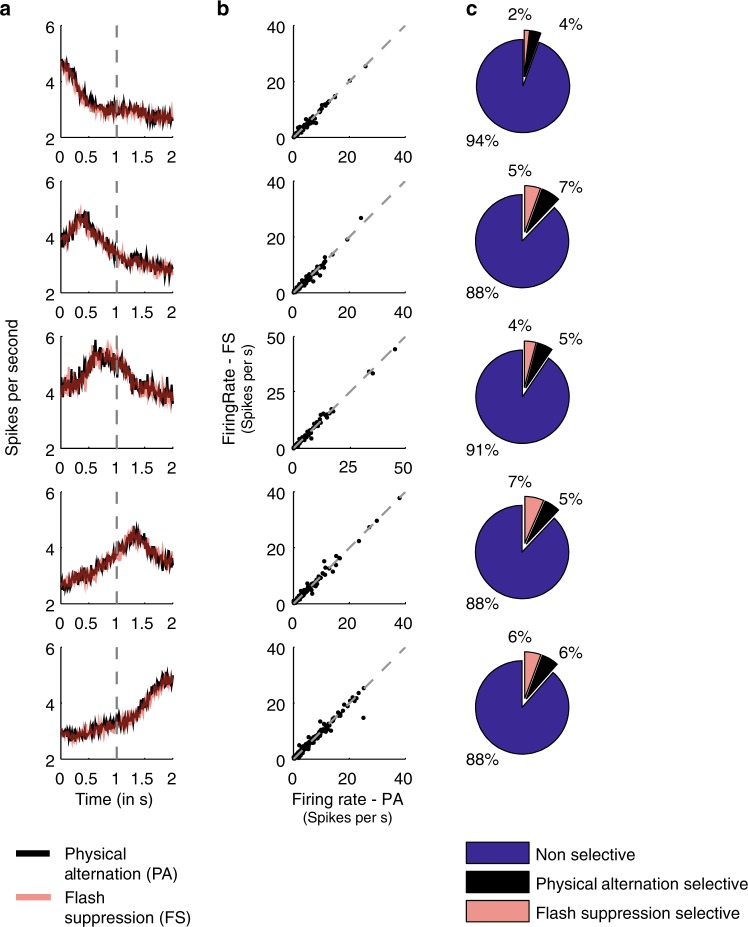
Single unit population activity. **a** Average population activity across all single units assigned similar to a given response profile in Fig. 3, during the condition of PA (black) and FS (pink). Note that the average activity is similar across the two conditions. **b** Scatter plot of the average firing rate of each single unit in the same cluster during the second part of the trial (1001–2000 ms). **c** Percentage of units (rounded to integers) which fired significantly higher in the physical alternation (black) or flash suppression (pink) condition. A majority of the units did not fire significantly different in either of the stimulus conditions (blue) (rank sum test, *p* > 0.05). Refer to the text for the exact number of units assigned to each CRP

We categorized the responses of individual units, by assigning them to the most similar CRP. Specifically, cosine similarity was used for comparing each single unit’s activity profiles to the CRPs obtained after NNMF-based decomposition. Each unit was thus sorted to one of the five patterns judged on the basis of maximum similarity to one of them (see Methods section). 356 units were assigned to CRP 1, 182 to CRP 2, 148 to CRP 3, 181 to CRP 4, and 216 to CRP 5. Next, in order to evaluate the impact of perceptually suppressed visual input on these response patterns, we quantified whether the single units sorted to each CRP displayed any differences in activity between monocular (PA) and binocular (FS) stimulation, when incongruent stimuli were presented to the two eyes resulting in perceptual suppression. Plotting the average single unit activity revealed a very similar response profile across the two conditions (Fig. 4a). A scatter plot of the average firing rates (time period—1001–2000 ms) of single units belonging to the same cluster is shown in Fig. 4b. Most of the units lie on the diagonal further exemplifying the similarity in their response across the PA and FS condition. Indeed, a majority of units (~90%) responded similarly in the two conditions, when their activity was compared during the second half of the trial (Fig. 4c). The total number of units assigned to the different CRPs which were not significantly different in their activity across the two conditions was: 315 for CRP1 (88%), 160 for CRP2 (88%), 134 for CRP3 (91%), 159 for CRP4 (88%), and 204 for CRP5 (94%) (rank sum test, *p* > 0.05). This result suggests that the presence of visual competition and suppressed visual information has a rather limited impact on sequential firing patterns in the LPFC.

### Sequential single unit activity spans the trial duration

**Fig. 5:**
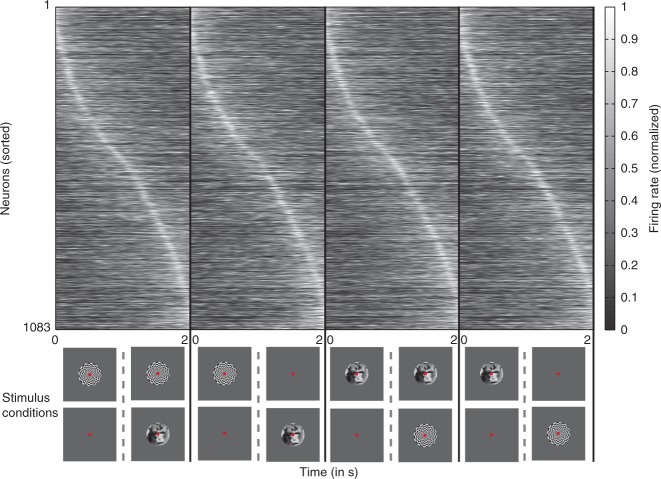
Single unit activity sorted according to the latency of the peak neuronal response. Each top panel displays the neuronal activity in the different stimulus conditions (shown in the bottom panel) sorted according to the latency of the peak amplitude. For determining the sorting order, the average PSTH across the four conditions was calculated, peak latency for the resultant PSTH was determined and neurons were sorted accordingly. Neurons tend to peak around the same time across the four stimulus conditions. Moreover, the peaks are distributed across the entire duration of the trial

Having observed that the five canonical responses spanned the entire duration of the trial, we next addressed whether the individual single unit activity also displayed such a sequential nature. Such successive neuronal activity has been typically observed among the rodent hippocampal cells signaling time and is known to be distributed across the entire duration under observation^33^. We first averaged the PSTH of each neuron (*N* = 1083) across the four experimental conditions, to obtain one activity profile per unit. The neurons were then sorted according to the latency of the peak of this average PSTH. Figure 5 shows the average PSTH activity (displayed as normalized firing rate) for each neuron according to the new sorting order in the four different conditions. The peak of neuronal responses was distributed through the entire duration of the trial signifying the sequential nature of the neuronal activity. Further, qualitative inspection of the activity revealed that it was very similar across the different stimulus conditions. It should be emphasized that CRPs obtained from NNMF are difficult to be inferred from a visual inspection of Fig. 5.

### Correlation structure of sequential neuronal activity

**Fig. 6:**
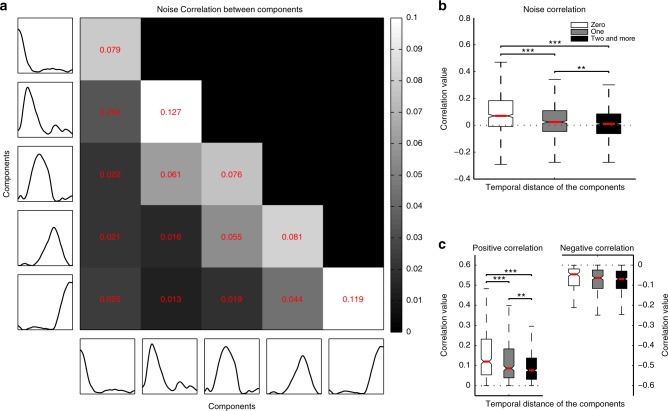
Noise correlation matrix and whisker box plots of correlations as a function of temporal distance. **a** Correlations were calculated across various combinations of neurons assigned to the sequential CRPs (arranged chronologically with earliest on the top and latest on the bottom along the y axis and earliest at the left most position and latest on the right most position on the *x-*axis). The brightness of the individual pixels depict the strength of correlation according to the colorbar on the right, with white and black depicting high and low correlations, respectively. Strong correlations were observed between neurons lying on the major diagonal, which constitutes contemporaneous neurons. The correlations decreased as depicted from the intensity of pixels across successive diagonals lateral to the main diagonal. Correlations in these consecutive diagonals are obtained from pairs of units classified to successive, temporally separated patterns (temporal distance of one, or two and more in **b** and **c** refers, respectively, to the first, or the second and the rest of the neighboring diagonals toward the left of the main diagonal). **b** Whisker box plots of noise correlations for different groups of neuronal pairs according to the temporal distance between the components they are clustered to. The red line denotes median, and the box denotes the 25th (Q1) and 75th percentiles (Q3) of the data. Contained within upper and lower whisker lengths are all adjacent values within Q3 + 1.5×(Q3−Q1) and Q1−1.5×(Q3−Q1), respectively. Notches approximate the 95% confidence interval around the median, and its edges are calculated as median ± 1.57  (Q3−Q1)/(square root of number of samples). Correlations decreased with an increase in the temporal distance between the CRPs to which the constituent single units were clustered to. **c** Whisker box plots of positive correlations displayed a trend very similar to the correlations in **b**. Whisker box plots of negative correlations which were not significantly modulated by temporal distance. In figures **b** and **c**, t-test (for sample numbers, refer to Supplementary Fig. 5) is utilized for comparison, *p ≤ 0.05, **p ≤ 0.01, ***p ≤ 0.001. See also Supplementary Fig. 6

**Fig. 7:**
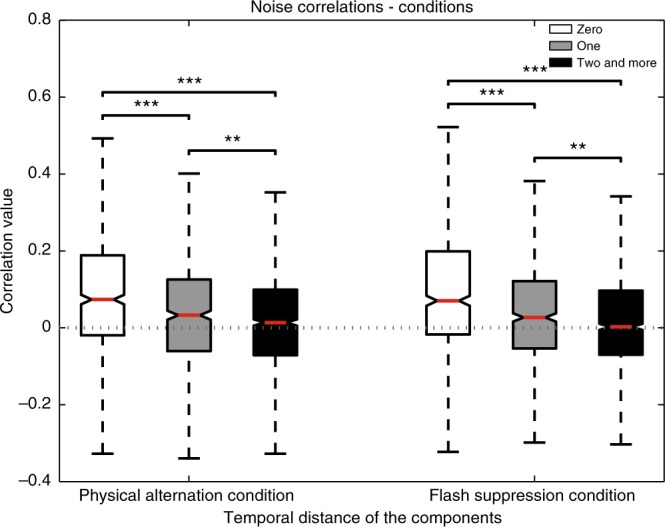
Noise correlations as a function of temporal distance for the PA and FS condition. The box plot referred to as zero, constitutes correlations among units clustered to the same CRP. Temporal distance of one, or two and more refers, respectively, to the first, or the second and the rest of the neighboring diagonals toward the left of the main diagonal as displayed in Fig. 6. Parameters for box plots similar to as in Fig. 6. Average noise correlations displayed a decrease as a function of temporal distance for both the PA as well as the FS conditions (t-test, *p ≤ 0.05, **p ≤ 0.01, ***p ≤ 0.001)

Single units displaying similar response types^34^ or high temporal rate correlation^35^ have been shown to display positive spike count correlation. Such pairwise correlated variability is thought to reflect direct or indirect synaptic connectivity or a shared common input and has been therefore used as a tool for functional segregation of neuronal populations^36–39^. In the visual cortex, such connectivity analysis has revealed that units with similar orientation preference display strong correlations and are functionally connected^28,40^. In this regard, we hypothesized that a plausible organizational principle for the prefrontal network showing sequential activity could rely on the temporal phase in which neurons were maximally activated. We sorted the CRPs chronologically, and calculated the trial by trial, spike count correlation across pairs of simultaneously recorded neurons (*N* = 2385 pairs), assigned to the different CRPs. The matrix displayed in Figure 6a depicts the average spike count correlations obtained across the various combinations of units categorized to the five different CRPs. The main diagonal includes all the neuronal pairs, wherein, the constituent units are clustered to the same pattern. This category is referred to as having a temporal distance (t.d.) of zero, or units clustered to the same CRP. Consecutive diagonals below the main diagonal display correlations, obtained from pairs of units classified to temporally separated CRPs (t.d. of one, or two and more refers, respectively, to the first, or the second and the rest of the diagonals below the main diagonal). Interestingly, correlations tend to be higher along the principal diagonal and decrease in successively lateral diagonals (histograms of the correlation coefficients, alongside the number of pairs for each comparison are shown in Supplementary Fig. 5). Specifically, the correlations between pairs of units with similar patterns were significantly stronger (mean *r*_sc_ (t.d._0_) = 0.1051 ± 0.0076 (S.E.M.)) than when the constituent units were from groups which were temporally divergent (mean *r*_sc_ (t.d._1_) = 0.0447 ± 0.0066, mean *r*_sc_ (t.d._2 and more_) = 0.0192 ± 0.0047; t-test (t.d._0_ vs t.d._1_), t-statistic = 5.93, *p* = 3.7×10^−9^; t-test (t.d._0_ vs t.d._2 and more_), t-statistic = 10.12, *p* = 1.8 × 10^−23^; t-test (t.d._1_ vs t.d._2 and more_), t-statistic = 3.1915, *p* = 0.0014; Fig. 6). Such a trend was also observed when correlations were computed for individual conditions, PA and FS separately (mean *r*_scPA_ (t.d._0_) = 0.1064 ± 0.0082, mean *r*_scPA_ (t.d._1_) = 0.0439 ± 0.0076, mean *r*_scPA_ (t.d._2 and more_) = 0.0198 ± 0.0051; t-test (t.d._0_ vs t.d._1_), t-statistic = 5.56, *p* = 3.2 × 10^−8^; t-test (t.d._0_ vs t.d._2 or more_), t-statistic = 9.42, *p* = 1.3 × 10^−20^; t-test (t.d._1_ vs t.d._2 and more_), t-statistic = 2.71, *p* = 0.0067; mean *r*_scFS_ (t.d._0_) = 0.1061 ± 0.0079, mean *r*_scFS_ (t.d._1_) *=* 0.0446 ± 0.0069, mean *r*_scFS_ (t.d._2 and more_) = 0.0193 ± 0.0053; t-test (t.d._0_ vs t.d._1_), t-statistic = 5.82, *p* = 7 × 10^-9^; t-test (t.d._0_ vs t.d._2 and more_), t-statistic = 9.4, *p* = 8.1 × 10^−21^; t-test (t.d._1_ vs t.d._2 and more_), t-statistic = 2.9, *p* = 0.0037; Fig. 7). Moreover, no significant difference was observed when the correlations obtained in PA were compared with FS at respective temporal distances (for all three t.d. (0, 1, and 2 and more) comparisons, t-test, *p* > 0.05), thus suggesting that the structure of functional connectivity is preserved irrespective of whether the visual input is monocular or binocular and involves perceptual suppression.

A previous study utilized a cross correlation analysis between pairs of neurons activated during different task epochs, which indicated that synaptic inhibition might play a role in mediating temporal information flow in the prefrontal cortex^41^. Since the area under the cross correlation curve is related to spike count correlation, we aimed at elucidating plausible excitatory and inhibitory interactions among neurons categorized to different CRPs by separating positive from negative noise correlations (Fig. 6c), respectively^28,42^. The positive correlations exhibited a trend very similar to the average correlation in that they displayed progressive decorrelation as a function of temporal distance (mean *r*_sc-pos_ (t.d._0_) = 0.1789 ± 0.0081, mean *r*_sc-pos_ (t.d._1_) = 0.1347 ± 0.0071, mean *r*_sc-pos_ (t.d._2 and more_) = 0.1115 ± 0.0052; t-test (t.d._0_ vs t.d._1_), t-statistic = 3.96, *p* = 8 × 10^−5^; t-test (t.d._0_ vs t.d._2 and more_), t-statistic = 7.18, *p* = 1.2 × 10^−12^; t-test (t.d._1_ vs t.d._2 and more_), t-statistic = 2.66, *p* = 0.0078). Surprisingly, the negative correlations were uniformly distributed across temporal distance (mean *r*_sc-neg_ (t.d._0_) = −0.0812 ± 0.0073, mean *r*_sc-neg_ (t.d._1_) = −0.0905 ± 0.0062, mean *r*_sc-neg_ (t.d._2 or more_) = −0.0935 ± 0.0046; t-test (t.d._0_ vs t.d._1_), t-statistic = 0.97, *p* = 0.3284; t-test (t.d._0_ vs t.d._2 and more_), t-statistic = 1.43, *p* = 0.1510; t-test (t.d._1_ vs t.d._2 and more_), t-statistic = 0.38, *p* = 0.7007). This correlation structure and the differences between positive and negative correlations (also see Supplementary Fig. 6) are reminiscent of recent results for sensory tuned neurons in the primary visual cortex^28^, suggesting that a similar structure of functional connectivity is relevant in neuronal populations encoding more abstract properties, like task phase in the LPFC. We should mention that the normalization of spike counts across trials, which is used to compute noise correlations removes the effect of mean neuronal responses. Furthermore, using the sum of spike counts across the total trial duration removes any contribution of temporal covariation.

### Functional segregation of task-phase and conscious content

**Fig. 8:**
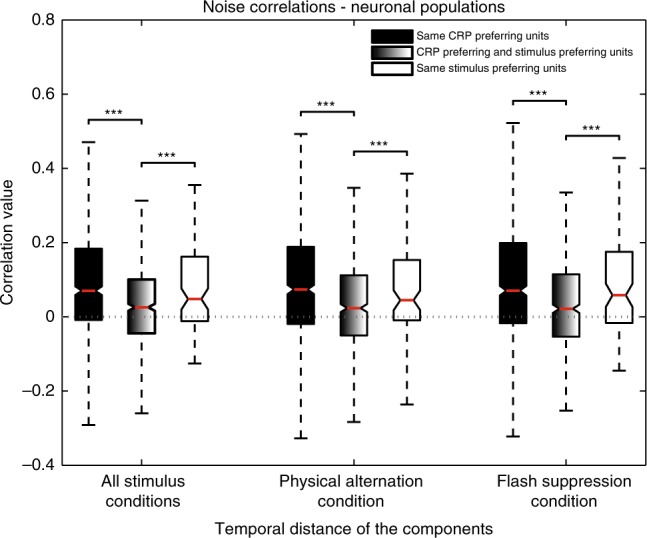
Noise correlations among pairs of single units belonging to the same neuronal populations or when each single unit comprising a pair belonged to different neuronal populations. The latter is displayed by the middle box plot for the various stimulus conditions (t-test, *p ≤ 0.05, **p ≤ 0.01, ***p ≤ 0.001). Parameters for box plots similar to as in Fig. 6. See also Supplementary Fig. 8

Lastly, given the ability of spike count correlations to indicate functional segregation of neuronal populations, we assessed if neurons categorized to the various CRPs are functionally segregated from the population of feature selective units that was described in detail in the past^11^ (Supplementary Figs. 1 and 7). As expected, pairs of units preferring the same stimulus (s-stim, *N* = 93 pairs) or categorized to the same CRP (s-crp, *N* = 688 pairs), displayed strong spike count correlation (mean *r*_sc-s-crp_ = 0.1051 ± 0.0076, mean *r*_sc-s-stim_ = 0.0891 ± 0.0155, Fig. 8). Interestingly, pairwise correlations between units belonging to these two different populations (crp-stim, *N* = 551 pairs) were dramatically and significantly lower (*r*_sc-crp-stim_ = 0.0374 ± 0.0052; t-test (s-crp vs crp-stim), t-statistic = 7.01, *p* = 3.9 × 10^−12^; t-test (s-stim vs crp-stim), t-statistic = 3.67, *p* = 2.5 × 10^−4^), thus suggesting functional segregation in their presynaptic inputs or decreased probability of direct or indirect intrinsic synaptic connectivity^36–39^. Further, such functional segregation was also observed for individual conditions (denoted by suffixes PA for physical alternation and FS for flash suppression), thus indicating that these two single unit populations remained functionally segregated irrespective of the input, whether it was monocular or incongruent (mean *r*_sc-s-crp-PA_ = 0.1064 ± 0.0082, mean r_sc-s-stim-PA_ = 0.0877 ± 0.0169, *r*_sc-crp-stim-PA_ = 0.0356 ± 0.0056; mean *r*_sc-s-crp-FS_ = 0.1061 ± 0.0079, mean *r*_sc-s-stim-FS_ = 0.0906 ± 0.0156, *r*_sc-crp-stim-FS_ = 0.0391 ± 0.0058; for both PA and FS: t-test (s-crp-PA vs crp-stim-PA), t-statistic = 6.76, *p* = 1.9 × 10^−11^; t-test (s-stim-PA vs crp-stim-PA), t-statistic = 3.4, *p* = 6.9 × 10^−4^; t-test (s-crp-FS vs crp-stim-FS), t-statistic = 6.55, *p* = 8.3 × 10^−11^; t-test (s-stim-FS vs crp-stim-FS), t-statistic = 3.31, *p* = 9.5 × 10^−4^. Moreover, such reduced functional connectivity between the two neuronal populations was also observed when the data from the three animals was analyzed individually (Supplementary Fig. 8).

Further, we exploited the NNMF procedure to decompose the stimulus selective neuronal population to obtain stimulus selective response patterns (SRPs) and computed the noise correlation between units clustered to different CRPs and SRPs (Supplementary Fig. 9). We observed a similar segregation of the two populations. Lastly, we used a spike width measure^43^ to compute the proportion of putative pyramidal cells (broad spiking neurons) and putative interneurons (narrow spiking neurons) in the different neuronal subpopulations in order to ascertain whether the two segregated populations corresponded to two distinct neuronal subtypes. This analysis revealed that their proportions were highly similar in the two functionally segregated neuronal populations (Supplementary Fig. 10).

## Discussion

We investigated the nature of non-feature selective neuronal responses in the LPFC during the no-report, passive fixation paradigm of BFS. We found task-phase related responses that were largely unaffected by visual competition and perceptually suppressed visual information. Importantly, these neurons were functionally segregated from neurons encoding the contents of conscious perception. We first discuss the impact of these results on the current debate regarding the role of LPFC in conscious perception, especially in the context of a no-report task. Thereafter, we discuss the relevance of these findings to temporally contingent neuronal activity in the LPFC.

Multiple theoretical models postulate PFC as a critical part of a widespread cortical network representing the contents of consciousness^16–18^. This has been cemented with evidence gathered from neuroimaging^18,44^, visual hallucinations induced by stimulation of the PFC^45,46^, impairment of conscious perception in macaques and human subjects following PFC lesions^21,47–50^, and more recently single neuron recordings^11,51,52,53^.

The clinical evidence however is debated because of reports describing patients with damage to the frontal lobe but intact conscious awareness^3,20,21^. Moreover, recent experimental work seems to suggest that frontal activity observed during paradigms investigating conscious perception is the result of task related demands or motor report^54–57^. Therefore, it has been advocated that no-report tasks are more appropriate for investigating conscious perception^12^. However, a lack of motor report does not preclude the absence of task related activity. Indeed, we found task-phase related sequential activity patterns among LPFC single units recorded simultaneously with units representing the contents of conscious perception in a no-report paradigm^11^. This finding suggests that different LPFC populations operate in parallel to encode the contents of consciousness and task phase.

Given that neuroimaging studies investigating conscious perception typically rely on a contrastive analysis in order to isolate neural signals mediating subjective perception from those merely related to the sensory input^44^, a difference in task related activity across control and experimental task conditions would be a potential confound in ascertaining the neural correlates of conscious perception. We find task-phase related activity at the level of single neurons was similar during the two trial types, and thus unaffected by sensory context. Additionally, the structure of correlations among population of units clustered to the various CRPs displayed an effect of temporal distance irrespective of the monocular and binocular input conditions, thus demonstrating a similar nature of functional interactions independent of the specific sensory input. Most importantly, spike count correlations were significantly stronger between pairs of units, which preferred the same stimulus or were categorized to the same CRP as compared to the correlations when each neuron comprising the pair came from two different populations suggesting that these neuronal pools are functionally segregated.

Since both task-phase and stimulus selective responses were observed in a no-report task of perceptual suppression, we believe that any additive influence of reporting for the percept would mostly affect the strength of selectivity in the stimulus selective population as shown previously^43^. Consequently, blood oxygen level dependent (BOLD) signal differences as measured with fMRI in the PFC between no-report and report paradigms^12,54,55^ could just reflect the additive influence of a motor component and the selectivity of neurons. A remarkable divergence between BOLD and the electrophysiological signals has been demonstrated in early visual cortex during perceptual suppression^58^. Similarly, our results demonstrate neuronal modulation related to perceptual content at the single unit level, supporting the involvement of PFC in conscious perception in contrast with the conclusions from fMRI studies.

NNMF-based decomposition revealed five dominant neuronal response patterns of temporally contingent neuronal activity in the LPFC. Tuning of neurons in this region to temporal order of events has been previously reported in the context of tasks, wherein sensory input dictated a requisite and sequential motor act^59–61^. Single neuron activity tracking task progress during a self-ordered task or of successively presented sensory stimuli as well as anticipatory activity related to expectation of reward has also been reported in the LPFC^62–64^. Additionally, sequential activity has also been observed in the frontal cortex of both primates and rodents engaged in various cognitive tasks^65–69^. Here, we similarly observed neurons that, sequentially increase their firing amplitude during different task phases in a no-report BFS task. The task only required animals to passively fixate without the need to carry out a serial motor action, but presumably did require monitoring ongoing task related behavior (that is, fixation), a key role attributed to the LPFC^24^. Taken together, the serial activity patterns observed in our data could encode the temporal phase or progress of the trial. Importantly, the observed sequential activity in the region remained unchanged during ambiguous stimulation suggesting that the underlying populations are unaffected by visual competition and presence of perceptually suppressed information. This further illustrates that such activity possibly subserves a more general monitoring function, in the absence of motor responses and irrespective of the nature of visual input and independent of the content of visual perception.

A key function of the primate LPFC, the temporal organization of behavior^25–27^ requires the successful parsing of the manifold sensory input into multiple sequential events in order to execute a temporally contingent motor response. It has been suggested that temporal segmentation of an ongoing visual input may happen automatically without an explicit instruction to do so^70,71^, and involves concomitant activation of the PFC, as observed in human BOLD fMRI recordings^72^. The LPFC sequential activity patterns during a no-report task without explicit instructions for segmentation of sensory input into events, indicates that prefrontal neurons are spontaneously organized in subpopulations, which may encode or tag specific periods of a repeatedly experienced perceptual task.

Interestingly, sequential activity patterns in the visual cortex have been observed in response to changing visual stimuli^73^. However, it remains unclear whether the activation related to temporal segmentation of ongoing visual input in the LPFC is an outcome of a passive feedforward integration of low-level features of the sensory signal or is governed by top-down influences of high level representation of a temporally contingent event^72^. If event related modulation among LPFC neurons was reliant on feature specific activity in sensory cortices, we would expect changes in their response during presentation of incongruent visual input. In contrast, the latter hypothesis suggests that the temporal segmentation or trial phase related activity in the LPFC should exhibit invariance to changes in the low-level sensory features. Specifically, we find that the nature of sensory input, whether monocular in case of PA, or dichoptic in case of FS, leads to very similar activity for a majority of the neurons (Fig. 4), providing evidence for the top-down influence hypothesis. This suggests that such sequential activity is independent of the retinal input related activity and might rely on the temporal percept of the subject.

Our results revealed that neurons with similar temporal profiles displayed the strongest correlations. It has been previously suggested that functional connectivity among PFC neurons could depend on their temporal correlation^74^ and such a relationship might be present even before task training^35^. Interestingly, we also observed these modulations among neurons recorded in the LPFC without any explicit mnemonic demands on the animal and for the first time in a task capable of disentangling conscious visual perception from sensory input. Such temporal dependency in correlation structure is presumably determined by the chronological structure of perceptual content. As the current percept and therefore the timing of perceptual events is unambiguously represented in the recorded region^11^, such temporal correlations can be expected to remain unaffected by ambiguous input. We observed a similar reduction in correlation as a function of temporal distance in both task conditions and the magnitudes of correlations for individual temporal distances were almost identical across conditions (Fig. 7). Thus, the organization of effective connectivity structure of neurons displaying task-phase related activity remained highly conserved across the two task conditions and was unaffected by suppressed visual input in a no-report paradigm.

Next, investigating the distribution of positive and negative correlations revealed that positive correlations displayed a decrease akin to the average correlations, but the negative correlations were uniformly distributed as a function of temporal distance. A dichotomy between positive and negative correlations was also reported in the visual cortex as a function of orientation difference^28^. Moreover, neurons in the visual cortex displaying correlation in their responses to visual stimuli have been demonstrated to exhibit strong and often reciprocal excitatory connectivity^75^. Similarly, sequential activity could be generated in a recurrent network model with dominantly excitatory synaptic interactions between temporally co-active neurons and reciprocal inhibition on temporally distant neurons^76^. In line with these observations, our correlation results suggest that sequential activity is implemented in the LPFC by excitatory connections between nearly contemporaneously activated neurons while inhibition is distributed evenly across all units.

This common correlation structure is observed among neuronal populations in different regions of the brain and involved in divergent neural processes. This may therefore reflect a general functional connectivity principle governing networks dedicated to the representation of sensory as well as more abstract features such as task-phase allowing an efficient segregation of functionally distinct subpopulations.

To conclude, the quest towards the physiological underpinnings of consciousness entails not only understanding how different brain regions orchestrate subjective experience but also delineating this participation from their concomitant roles in mediating other cognitive functions. While no-report tasks have been proposed to circumvent these issues, their limitations and advantages need to be further scrutinized. The results presented here suggest the presence of task-phase related activity even during the no-report task of BFS, albeit maintained across experimental conditions. BFS is a variant of the binocular rivalry paradigm, wherein the former, the perceptual switches are externally controlled while in the latter, these transitions happen internally without a change in external stimulation. Whether and how dynamics of such task-phase related activity could exist in a free flowing no-report binocular rivalry task remains to be investigated.

## Methods

### Behavioral task and stimulus presentation

The task consisted of two stimulus conditions, namely, physical alternation (PA) and flash suppression (FS), which were pseudo randomly interleaved (Fig. 1). Each trial in both conditions started with the presentation of a fixation spot (foveal presentation, size: 0.2°). Three hundred milliseconds later, a monocular visual stimulus (size: 2–3°) was presented (overlaid by the fixation spot) for 1000 ms. In PA trials, 1000 ms after the presentation of the first stimulus, this stimulus was removed from the first eye, and a second visual stimulus in the corresponding location of the other eye was presented. During FS trials, this disparate visual stimulus was presented without the removal of the first visual stimulus from the first eye. This results in the robust perceptual suppression of the stimulus presented first^11,13,14,30^. Therefore, the two different trial conditions were identical perceptually but differed in the concomitant sensory input during the second half of a trial (wherein a monocular stimulus without competition was present during PA trials or a suppressed stimulus was physically present during FS trials). The stimulus order and eye (in which the first stimulus was presented) was pseudo randomized and balanced across trials in a single dataset. Animals were trained to limit their fixation within a fixation window (±1°) for the entire duration of a trial and following successful fixation were given a liquid reward. Eye movements were monitored throughout the behavioral and electrophysiological recording and stored offline for further analysis. All the visual stimuli were presented with the help of a stereoscope and displayed at a resolution of 1280 × 1024 on the monitors (running at a 60 Hz refresh rate) using a dedicated graphics workstation (TDZ 2000; Intergraph Systems, Huntsville, AL, USA). Animals sat in a custom designed chair during the behavioral training and electrophysiological recordings.

### Electrophysiological recordings

Three healthy male adult rhesus monkeys (Macaca mullatta), D’98, F’03, and F’06, aged 14, 10, and 9 years, respectively, participated in electrophysiological recordings. All experiments were approved by the local authorities (Regierungspraesidium) and were in full compliance with the applicable guidelines of the European community (EUVD 86/609/EEC) for the care and use of laboratory animals. The cranial headpost and recording chamber were custom designed for each animal based on the high resolution MR scans collected using a 4.7 Tesla scanner (Biospec 47/40c; Bruker Medical), while the animal was under general anesthesia. The MR scan was also used for localizing the region of interest and the chambers were implanted over the LPFC. Implantation of the recording chambers was carried out under aseptic conditions as reported previously^77^. Additionally, a scleral eye coil was also implanted for measuring eye movements.

Custom made nichrome wire tetrodes (electroplated with gold for reducing impedance) were loaded on a custom designed microdrive for recording the neural activity in the LPFC. Cheetah data acquisition system (Neuralynx, Tuscon, AZ) was used to acquire, amplify, filter, and store the extracellular signal recorded with the tetrodes. The signal was sampled at 32kHz and digitized to 12 bit precision. Multi-unit spikes were detected by finding the time points at which the signal filtered in the 0.6–6 kHz range exceeded a predefined threshold (typically 25 μV). Spike sorting for single units was done offline using custom algorithms, which utilized the first three principal components of the spike waveforms as features^78^. Cluster cutting was performed using Klusters^79^.

### NNMF-based clustering of single unit activity

Neural data was recorded from 1285 neurons. 151 units displayed statistically significant stimulus selectivity in their response to one of the two presented stimuli during the PA version of the task (period compared: 1001–2000 ms, Wilcoxon rank sum test, *p* < 0.05). These units (~12% of the total), which displayed sensory selectivity, were removed thus leaving a total of 1134 neurons. We aimed to cluster this neuronal activity with a non-negative matrix factorization (NNMF) method^31,32^. The data, which was input to the algorithm, was preprocessed in the following way. First, the trial averaged peri stimulus time histogram (PSTH) from time 0 (first stimulus on) to 2000 ms (stimulus off), smoothed using a Gaussian kernel of standard deviation—40 ms, was computed for each single unit recorded. Next, the four PSTH’s per unit for four different conditions thus obtained were concatenated one after the other giving in all 1004 (251 per condition) data points per neuron. The order of the conditions was as follows: (a) Flash suppression (polar stimulus first and face stimulus second), (b) physical alternation (the same order as in (a)), (c) flash suppression (face stimulus first and polar stimulus second), and (d) physical alternation (the same order as in (c)). We removed from this matrix, all units with very low spiking activity (leading to the removal of units with an average of <1.26 spikes per concatenated trial; 1083 units passed the aforementioned criterion). Since a preliminary scrutiny of the unit response patterns revealed that most units were spiking in a relatively small portion of the trials, we aimed at finding automatically in the data, a set of non-negative canonical neural responses. We thus chose an NNMF-based model to decompose the recorded units as a mixture of canonical firing patterns with non-negative coefficients. We argue that such a decomposition in this context is more natural than a PCA-like approach that would lead to components with both positive and negative responses along the trials, and thus would be difficult to interpret individually as a unit response pattern. In contrast, each of the components found by NNMF can be interpreted as one “typical” response pattern found in the population, assuming it approximates well the PSTH of a subset of units in the analyzed population (this assumption was supported by our results). As a consequence, we assume that the unit response matrix **R** with dimensions (1083 (number of units) × 1004 (time points)) can be decomposed as a matrix product:

$${\mathbf{R}} \approx {\mathbf{HW}}$$

where, **W** is the (*K*  × 1004) matrix gathering the dominant *K* canonical temporal response patterns observed in the data, and **H** is a (number of units × *K*) weight matrix gathering the weights associated to the contribution of the *K* canonical response patterns to each unit response. The factorization was initialized by drawing dominant temporal responses at random using independent identically distributed coefficients (drawn as the absolute value of standard Gaussian, to which an offset of 0.1 is added to avoid initializing to values close to zero), and the corresponding time contributions were initialized using least square regression. Since the resulting regression coefficients are not guaranteed to be non-negative, we then kept only the positive part of each entry to build the initialization matrix and added a small positive quantity (2.22 × 10^−16^). The NNMF decomposition was then optimized using the multiplicative update algorithm^80^ and the stability of the solution was enforced using an iterative bootstrap approach described in the following paragraph.

For each initialization, we ran 50 bootstrap iterations as follows. Starting from the initialized matrices, we partitioned the rows of the matrix **R** in two subsets of equal length by random permutation of the rows, the first half of permuted rows being assigned to the first subset and the rest to the second. On the first iteration, the NNMF optimization was run separately on the two matrices built from the rows of the respective subsets, both using the initialization matrices described above, resulting in two sets of canonical responses. We reasoned that both solutions should in principle reach similar values for **W,** assuming that the number of collected units is large enough in order for both subsets to faithfully capture the variability of responses in this cortical region and that the solution of the NNMF is stable for the assumed number of response patterns *K*. To enforce such stability, pairs of canonical responses obtained by both optimizations are compared according to a cosine similarity measure. For pairs exceeding the similarity threshold of 0.5, the corresponding rows were retained for the initialization of **W** at the next bootstrap iteration, while the remaining rows were simply re-initialized again using random values, and initialization of **H** by linear regression followed, as described above. After 50 bootstrap iterations, we exploit the components found in the last 15 iterations to analyze the stability of the algorithm as follows. First, the average of these components (across iterations) is used to build the final initialization matrix and the NNMF algorithm is run one last time on the full response matrix **R** to generate the final solution. Second, the variance across the last 15 bootstrap iterations is used for quantifying the stability of the canonical responses. Since the order of canonical responses at each bootstrap iteration might change, we first reordered the *K* components of each iteration so that they match together. The ordering of the components was done by computing the kernel principal component analysis (KPCA) of all rows of both the **W** matrices pooled together. Then, the rows of each matrix were reordered according to the value of their projection on the first KPCA component. Once the components were reordered, the canonical response variability was then computed as the ratio of each (reordered) canonical response variance across the last 15 iterations of the NNMF algorithm to the corresponding canonical response mean square (both quantities being averaged across time).

We determined the optimal number of NNMF canonical responses *K* by running the previous procedure in 100 runs each for *K* varying between 2 and 10, and thereafter evaluated for each run and each *K*, the average across components of the canonical response variability ratio defined above. We selected the optimal *K* as the largest number of components achieving median component variability across runs inferior to 5% and for this value of *K* the NNMF solution achieving minimum residual error among all runs, defined as the entry wise sum of squares of the difference between the unit response matrix and its approximate factorization

$$\sum\limits_{u \in {\mathrm{units}}} \,\sum\limits_{t \in {\mathrm{time}}\ {\mathrm{samples}}} \left( {\left( {\mathrm{R}} \right)_{u,t} - \left( {{\mathrm{HW}}} \right)_{u,t}} \right)^2,$$

was selected as the optimal one. To assess the reliability of the selection of *K*, the whole procedure was run 10 times, such that the fluctuations of the canonical response variability as a function of *K* can be visualized (Supplementary Fig. 4). To quantify how well the NNMF decomposition with *K* components is approximating the data, we also computed the explained energy: the ratio of the entry-wise sum of squares of the factorization **HW** to the sum of squares of the unit response matrix **R**. Empirically, this proportion happens to be complementary to the entry-wise sum of square of the residual error introduced above (also taken relative to the sum of squares of **R**), following the same intuitive idea as variance decomposition in PCA analysis.

### Noise correlation analysis

The spike count correlation coefficient (*r*_sc_) was calculated for pairs of neurons during the time window of visual stimulation (temporal duration—2 s) as follows. First, we normalized the total number of spike counts for every trial of each single unit constituting the pair of neurons (*i* and *j*) by converting them into *z*-scores. This was done for each of the four stimulus conditions separately. Thereafter, the *z*-scores thus obtained across conditions were concatenated to obtain two vectors, respectively, for the pair of units. Next, the Pearson’s correlation coefficient was computed for the two vectors **z**_**i**_ and **z**_**j**_ using the corr function in MATLAB (MathWorks, Natick, MA, USA). The lack of variance among the *z*-scores of one or both the single units, resulted in a NaN output. These pairs did not contribute to the average correlation values.

### Statistical information

All statistical analysis was performed using MATLAB (MathWorks, Natick, MA, USA). We tested the stimulus preference of 1285 single units recorded from the LPFC of three monkeys. This was judged by comparing the spike responses in the two different types of PA trials as exemplified in Fig. 1a. We collated trials across conditions where the stimulus order was the same, although the visual stimulation started with two different eyes and then made this comparison. We contrasted the spike responses elicited during the second half of these trials with a two-tailed rank sum test. We also tested if the population of single units (*N* = 1083) whose response profiles were decomposed using the NNMF analysis, displayed any significant difference in their activity during monocular and incongruent visual input. This was judged by comparing the spike responses during the second half of PA and FS trials with a two-tailed rank sum test. Results are displayed in Fig. 4.

With respect to the individual correlation coefficients between pairs of neurons, we tested if they were significantly different from zero using the corr function in MATLAB (MathWorks, Natick, MA, USA), which outputs the *p*-values for the Pearson’s correlation coefficient. This is computed using a Student’s t-distribution for a transformation of the correlation. All statistics comparing the distribution of correlation coefficients among different neural subpopulations (Fig. 6b and c, Fig. 7, and Fig. 8) were carried out with a two sample unpaired t-test. All statistical analysis utilized a *p*-value ≤ 0.05 for determination of significance.

### Code availability

The code for NNMF analysis is available on figshare.

## Electronic supplementary material

Supplementary Information

## Data Availability

The data consisting of the response matrix (of single unit PSTHs) are available on figshare.
