# Supplementary material for: Parallel and functionally segregated processing of task phase and conscious content in the prefrontal cortex
Source: Commun Biol. 2018 Dec 5;1:215. doi: 10.1038/s42003-018-0225-1 (PMC6281663; doi:10.1038/s42003-018-0225-1)
Supplement: Supplementary file 1 — Supplementary Information [file 42003_2018_225_MOESM1_ESM.pdf]

SUPPLEMENTARY INFORMATION

SUPPLEMENTARY FIGURES

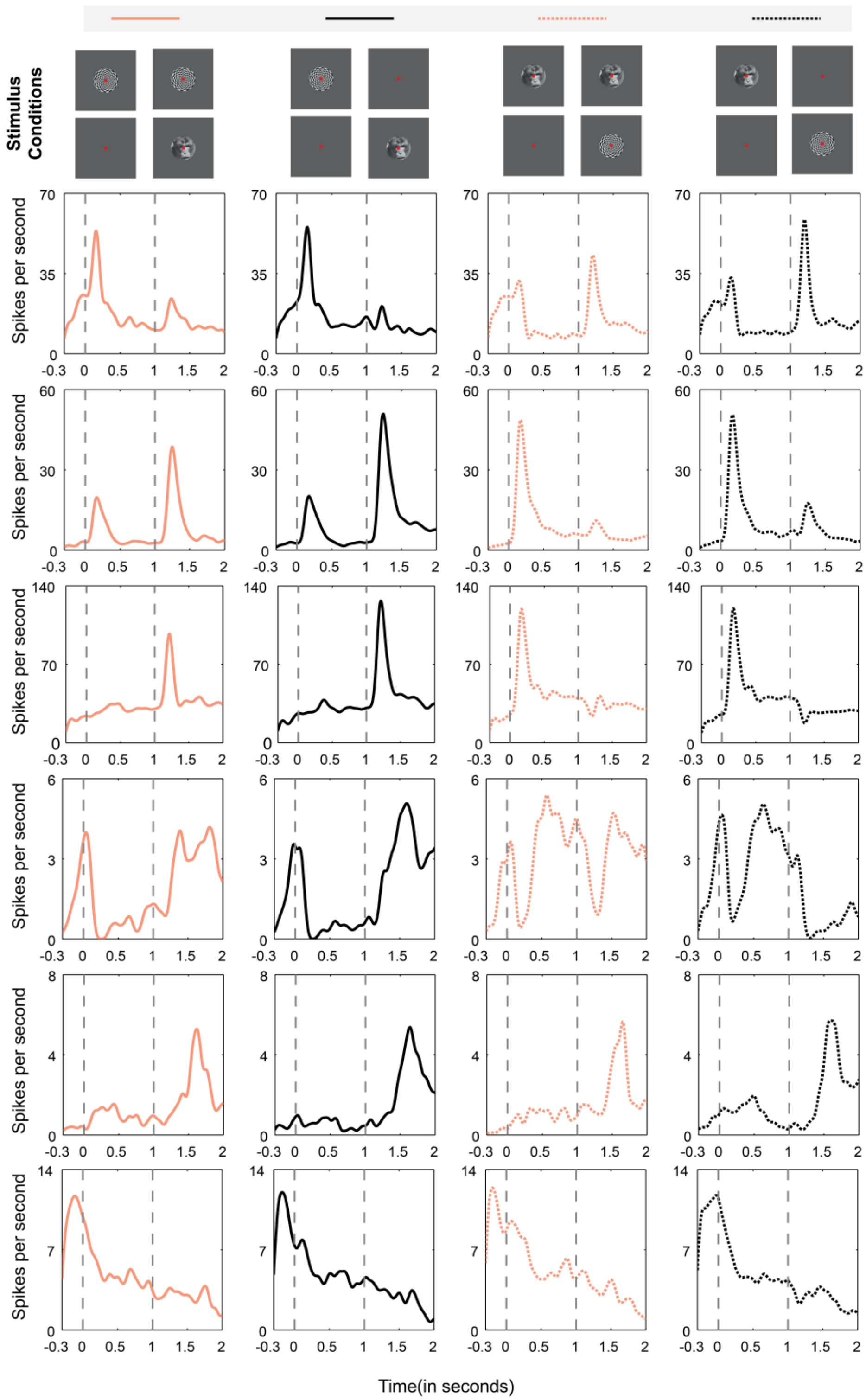

Supplementary Figure 1: **Example single unit responses of different neurons recorded during PA (black) and FS (orange).** Displayed are PSTHs of six example neurons. Solid lines indicate the neuronal responses when checkerboard pattern is presented first followed by the face stimulus. Neuronal responses when the face is presented first followed by the checkerboard pattern are plotted with dotted lines. The stimulus conditions are also displayed in the uppermost panel. In the first row is plotted example activity of a neuron which displayed preference to the checkerboard stimulus. The next two rows display example neurons selective for the face stimulus. In the fourth row is an example neuron which is selective in the PA condition, but loses selectivity in FS condition during the second half of the trial. Plotted in the last two rows is the PSTHs of two task phase related units for a comparison of the time course of their activity with that of feature selective units.

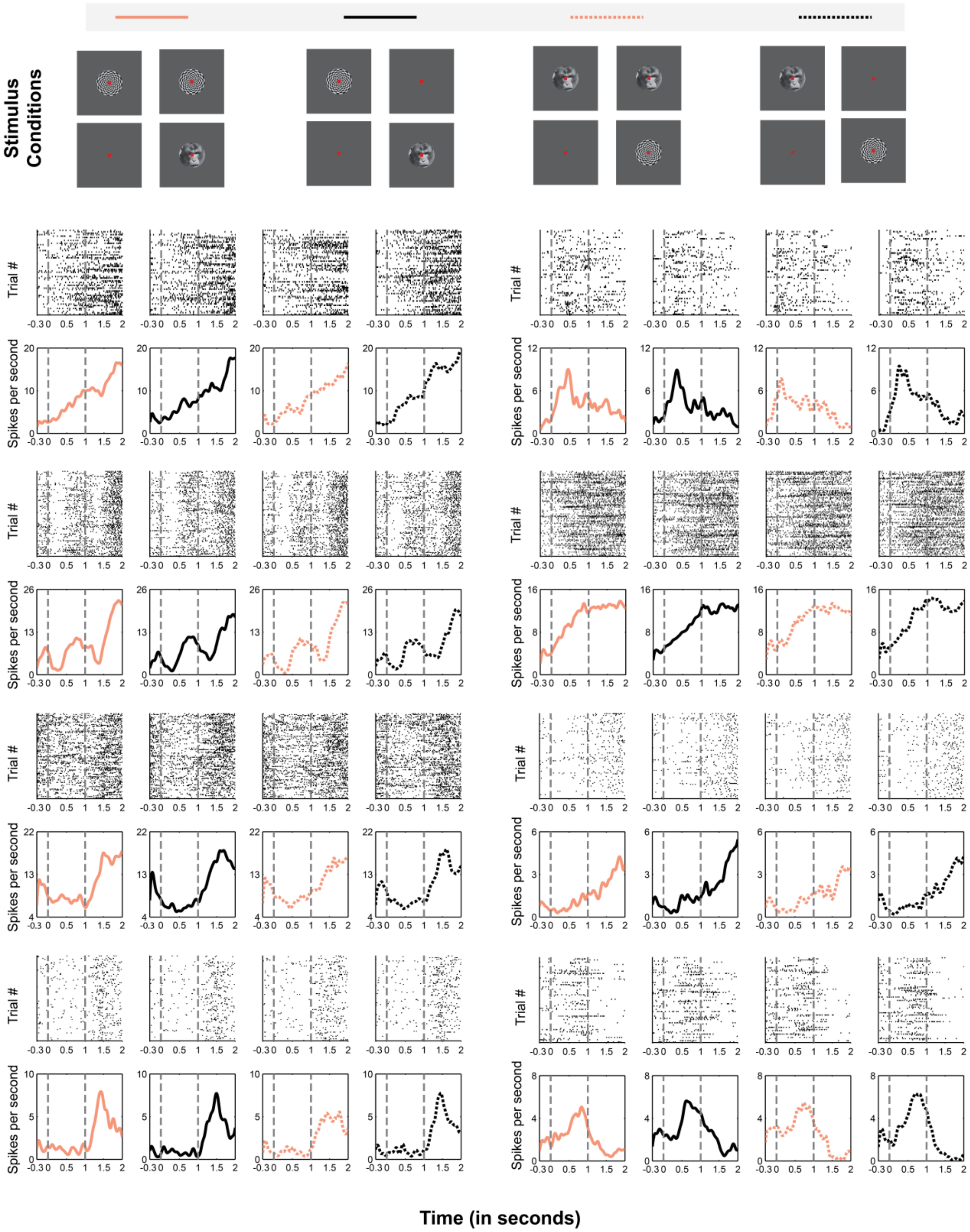

Stimulus Conditions
Trial #
Spikes per second
Time (in seconds)

Supplementary Figure 2: **Additional examples of single unit responses recorded during PA (black) and FS (orange).** The format of the figure is similar to figure 1 in the main manuscript. Displayed are each neuron’s single trial response (Trial # refers to different trials) as raster plots. Below the raster plots is the PSTH of each neuron. A considerable heterogeneity is observed among the response patterns of different neurons, with some cells peaking in an earlier phase of the trial and some later. (Line color and style conventions similar to Supplementary Figure 1).

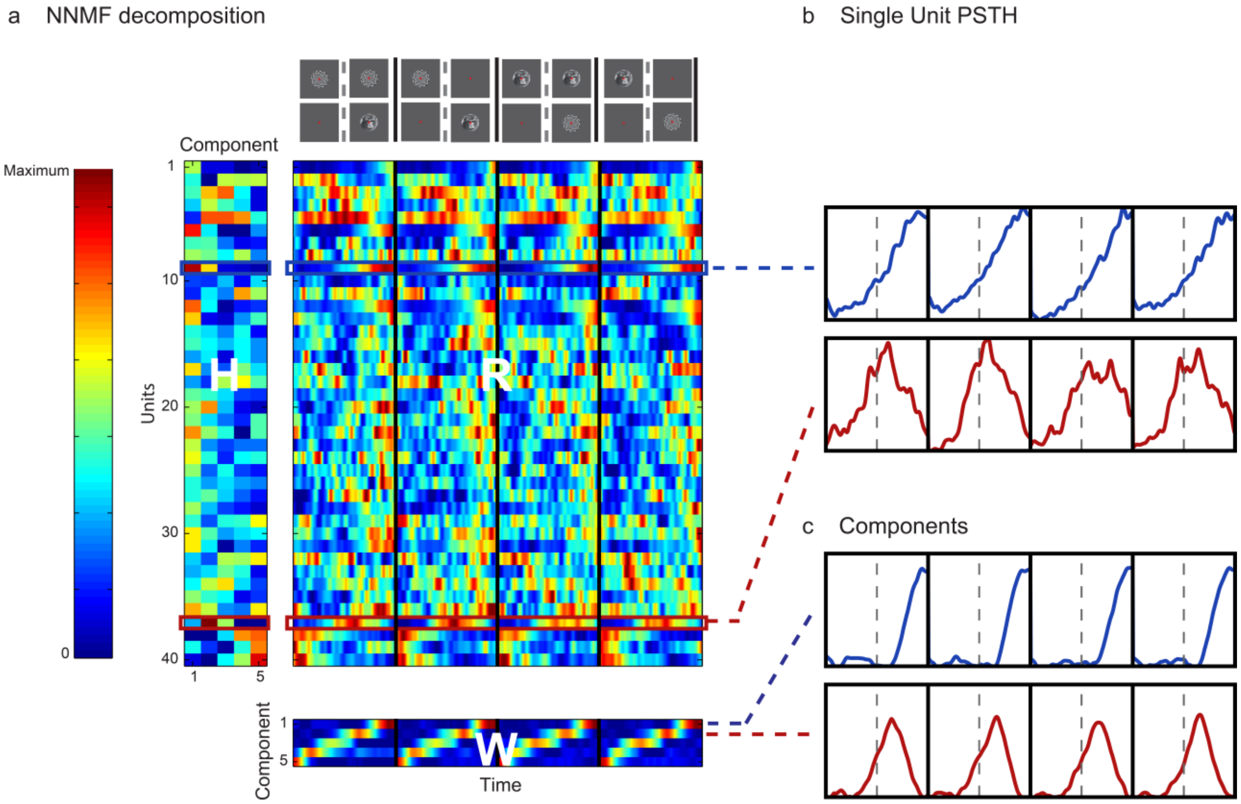

Supplementary Figure 3: **Principle of the NNMF decomposition.** (a) The matrix of single unit average response R (center) is decomposed into the product of two non-negative matrices: W the matrix of the time varying response of each component (displayed below the R matrix) representing typical response patterns for task phase related units, and H the matrix gathering the contribution of each component to a given unit response. (b) Two example single unit responses (in blue and red), each of which has a dominant coefficient in the corresponding row of the matrix H in panel (a), corresponding to component 1 and 2 respectively. (c) Time course of the two components corresponding to the dominant response pattern of the respective single unit responses shown in panel (b).

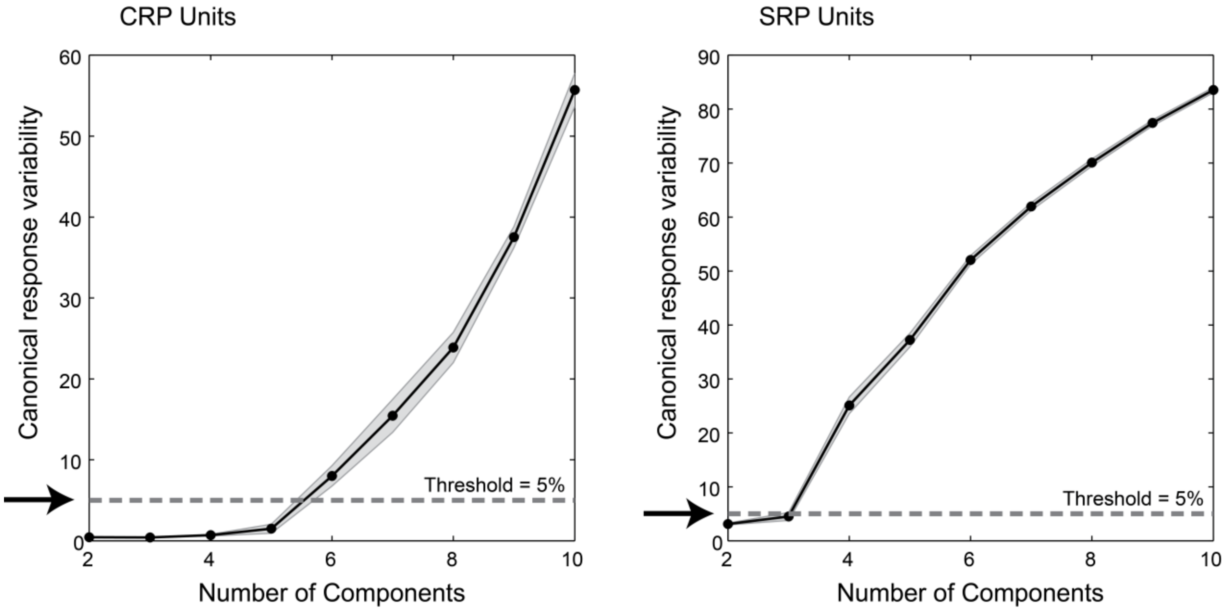

Supplementary Figure 4: **Canonical response variability estimate utilized for the choice of number of components.** An estimate of the canonical response variability is plotted as a function of the number of components chosen for the NNMF procedure. The standard deviation is across 10 different runs. The threshold is set at 5 percent, which directed our final choice for the number of components. Displayed on the left and right is the variance estimate when the NNMF procedure was carried out on task-phase related units and stimulus selective unit population respectively. Please refer to the methods section for details.

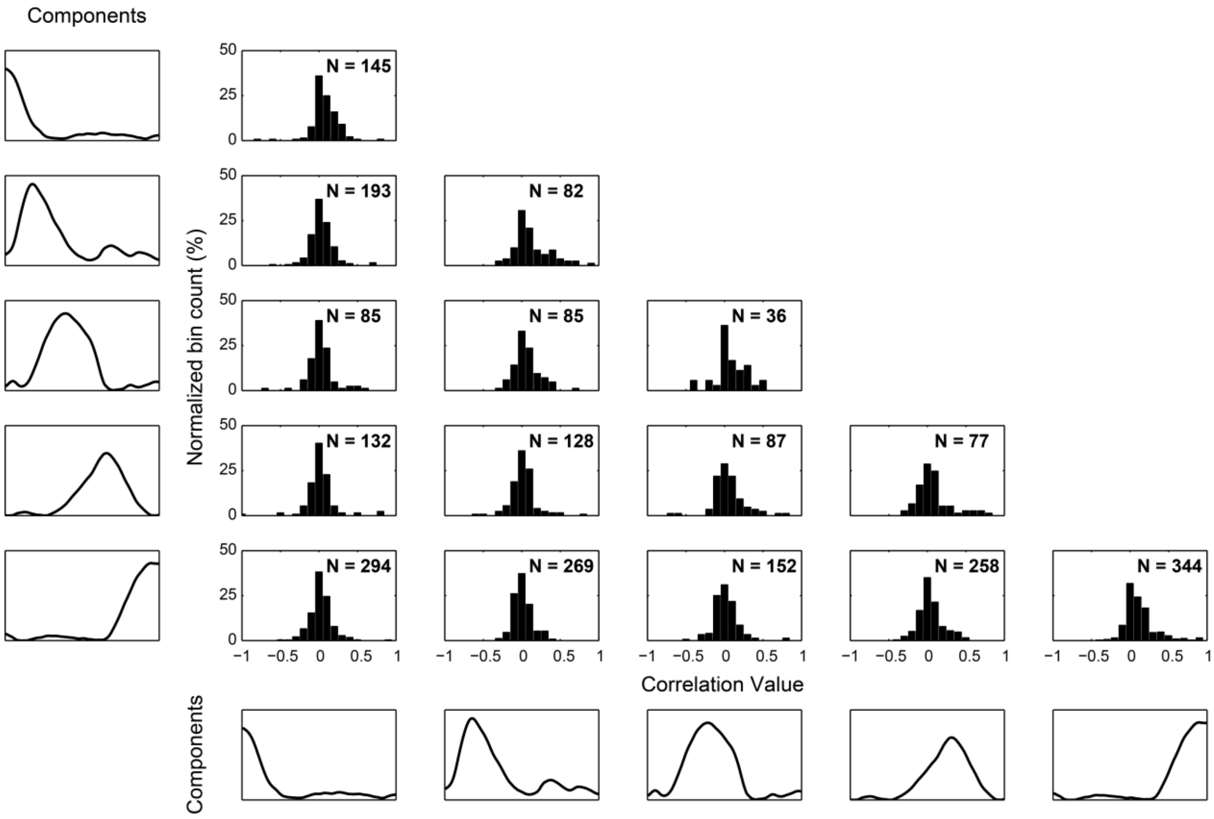

Supplementary Figure 5: **Noise correlation histograms.** Displayed are histograms which contribute to the average correlations presented as single pixels in the noise correlation matrix shown in figure 6 (a) in the main text. 'N' refers to the number of neuronal pairs. Upon a closer inspection of the various histograms along the major diagonal, one observes a bias in the distribution towards correlation values greater than zero. The panels displaying the histogram of correlations across successive lateral diagonals show peaks of the distribution, closer to zero.

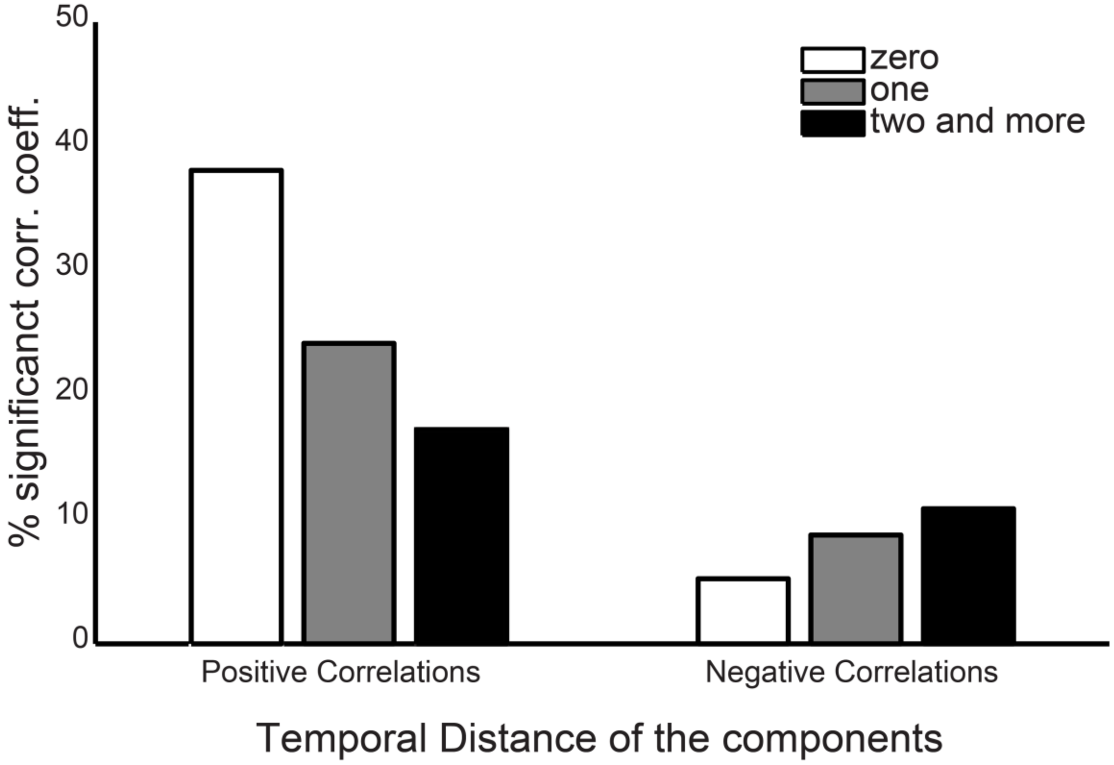

Supplementary Figure 6: **Percentage of significant positive and negative noise correlations among neuronal pairs as a function of temporal distance between the CRPs they are sorted to.** The proportion of significant positive noise correlations steadily decreased as a function of temporal distance. Moreover the proportion of significant positive noise correlations was ~25 percent compared to ~8.6 percent significant negative noise correlation. Such a dichotomy in fraction of significant positive and negative noise correlations has also been observed among neurons in the primary visual cortex, as a function of tuning similarity[1].

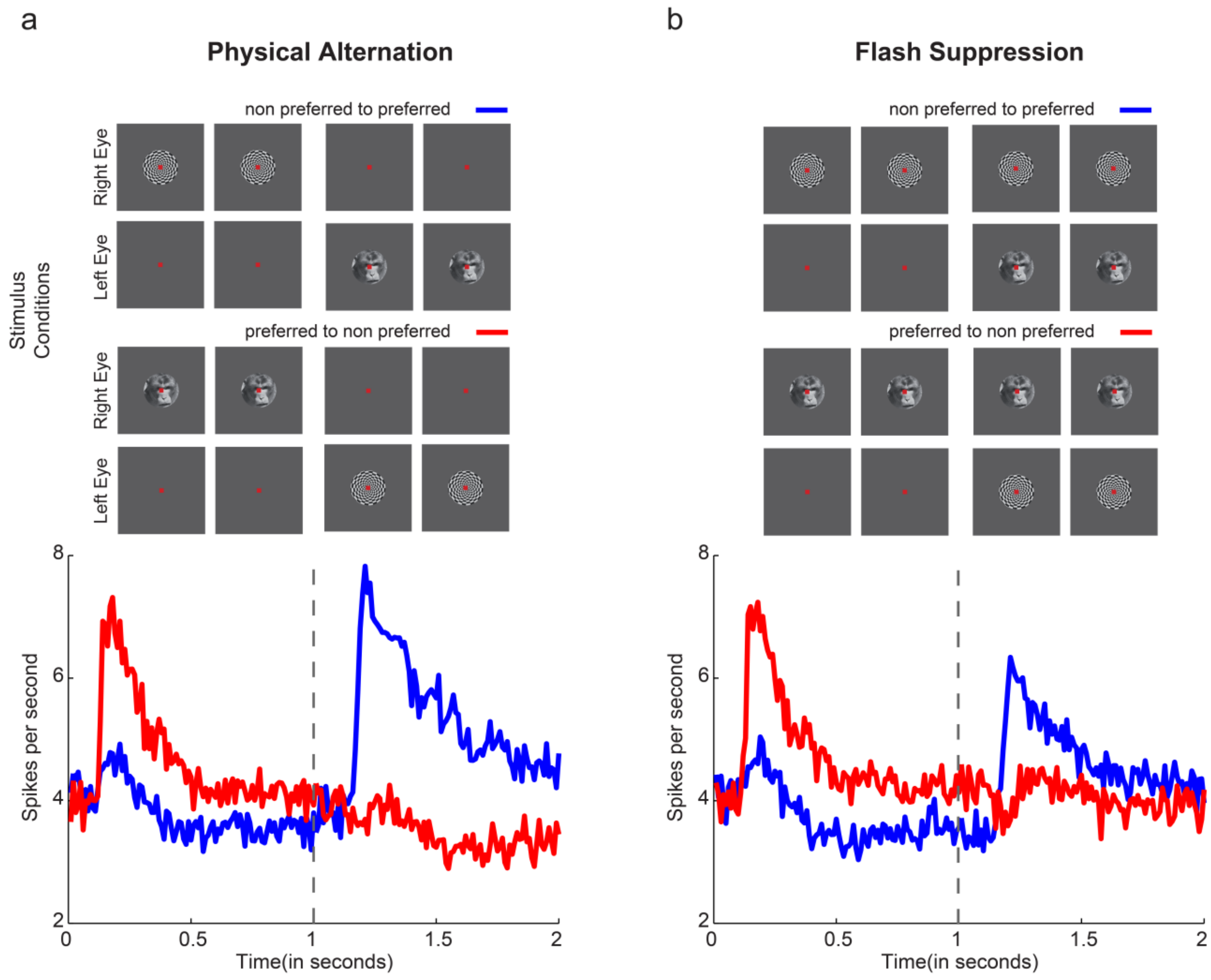

Supplementary Figure 7: **Population activity of visually selective neurons during PA and FS.** (a) Average population single unit activity of all neurons displaying significant stimulus preference in the PA condition of the task (N = 151). Red curve depicts the average single unit activity when neuron's preferred pattern was presented first, followed by the non-preferred pattern to the contralateral eye. Blue curve depicts the average single unit activity when visual stimulation started with the non-preferred pattern followed by neuron's preferred pattern. (b) Same as (a) for FS condition.

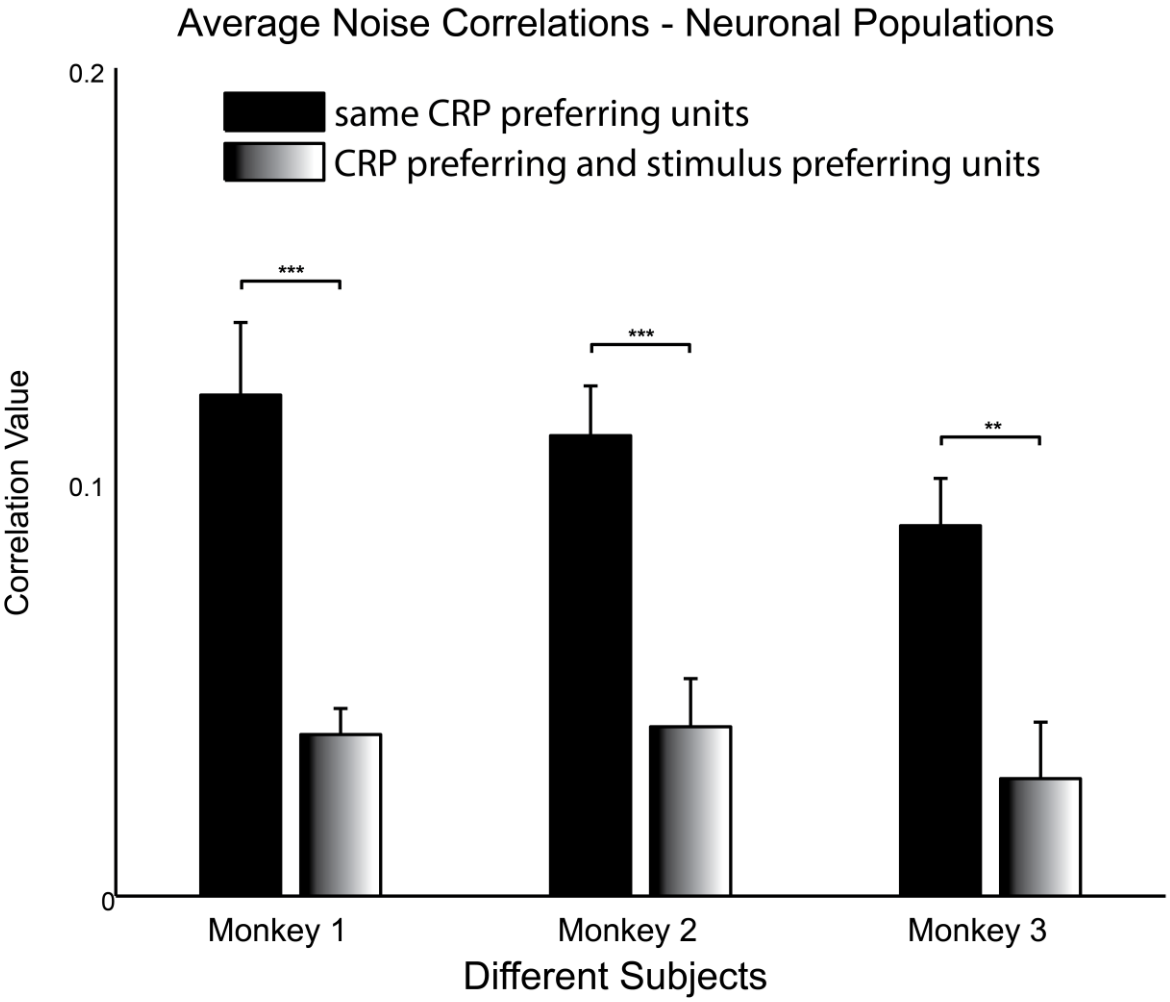

Supplementary Figure 8: **Noise correlations among different neuronal populations for the three different animals.** Average noise correlations among pairs of single units preferring the same CRP were significantly stronger as compared to when each single unit comprising a pair belonged to different neuronal populations. Such functional segregation was observed among single units recorded in all three different animals individually (t-test, ** - $p \leq 0.01$, *** - $p \leq 0.001$).

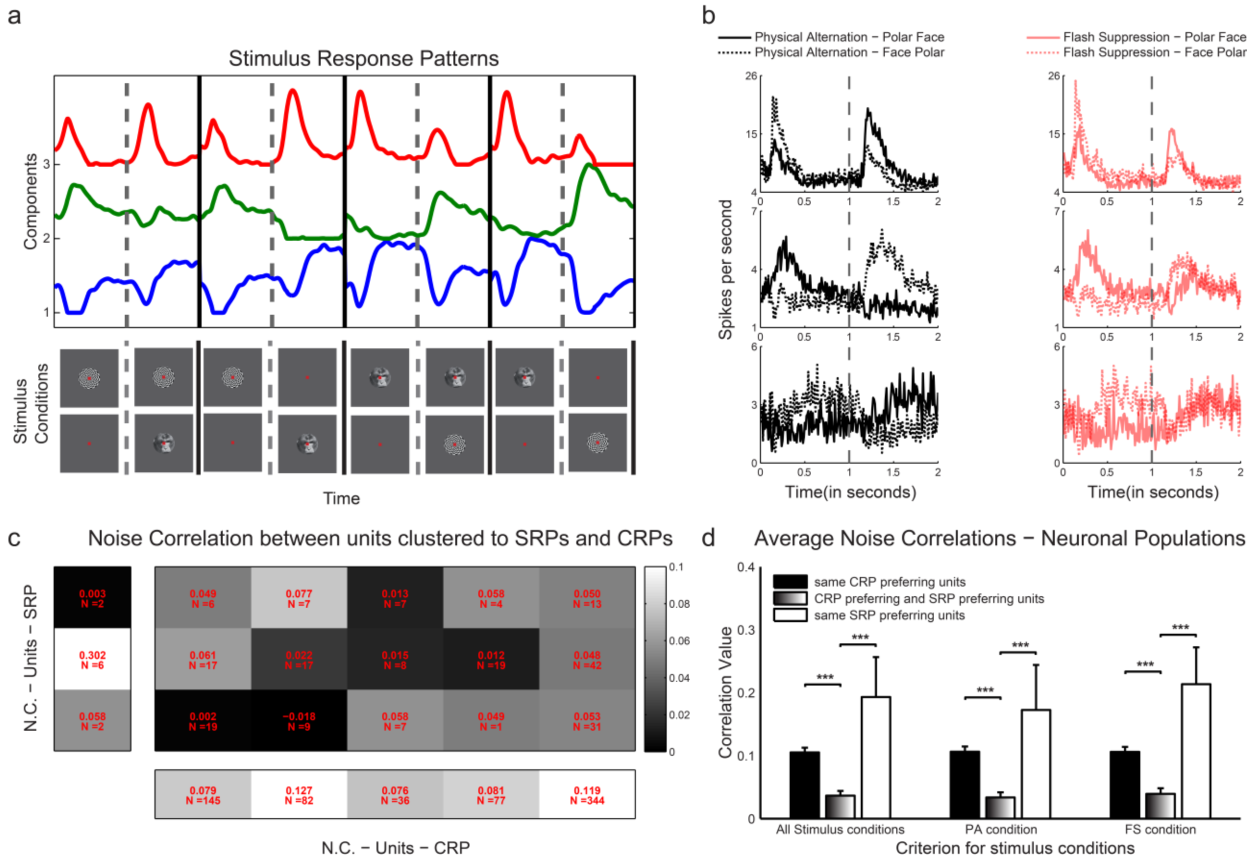

Supplementary Figure 9: **Stimulus-selective response patterns (SRPs) obtained after NNMF based decomposition of the feature selective neural responses and the structure of their correlations with task phase related neuronal population.** We removed from the stimulus selective single unit population (N = 151), any units which displayed rank sensitivity (stronger response during the first or the second half of the trial during PA condition), and decomposed the PSTHs of the rest of the units (N = 65 after removing low spiking units) with the NNMF procedure. (a) Three stimulus selective response profiles (SRPs) were obtained. Two response patterns, component 2 and 3 displayed stronger response for polar and the face stimulus, respectively. The SRP showed

in blue displayed selective responses in the PA condition, but lost this preferential activity during FS condition. The lower panel displays the stimulus presentation conditions, demarcated by black lines. (b) Average population activity across all single units assigned similar to a given response profile in (a), during the condition of PA (black) and FS (pink). The solid and dotted lines refer to the two different stimulus order. (c) Noise correlation matrix between units clustered to SRPs and CRPs. Correlations were calculated across neurons assigned to the sequential CRPs (arranged chronologically with earliest on the left and latest on the right along the x axis) and to the SRPs. Individual pixel brightness depicts strength of the correlation with white and black depicting high and low correlations respectively. Strong correlations were observed between neurons clustered to the same CRP or the same SRP (however, note the relatively low number of pairs (denoted by N) for SRP). The correlations seem to decrease as depicted from the intensity of pixels when one of the units comprising a pair was clustered to a CRP and the other to an SRP. (d) Average noise correlations among pairs of single units belonging to the same neuronal populations were significantly stronger, compared to when each single unit comprising a pair belonged to different neuronal populations (t-test utilized for comparison, * - $p \leq 0.05$, ** - $p \leq 0.01$, *** - $p \leq 0.001$).

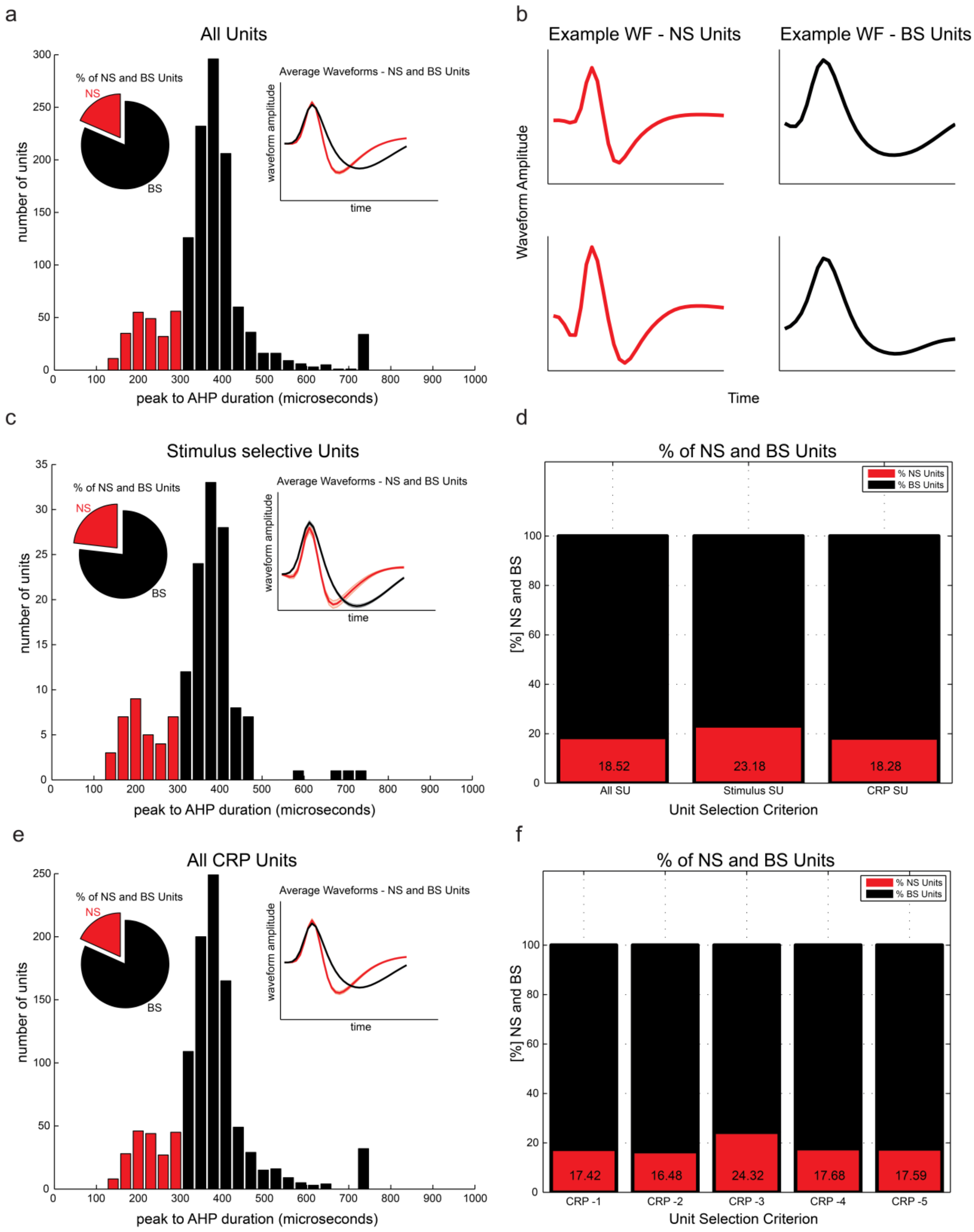

a
All Units
% of NS and BS Units
NS
BS
Average Waveforms - NS and BS Units
waveform amplitude
time
number of units
peak to AHP duration (microseconds)
b
Example WF - NS Units
Example WF - BS Units
Waveform Amplitude
Time
c
Stimulus selective Units
% of NS and BS Units
NS
BS
Average Waveforms - NS and BS Units
waveform amplitude
time
number of units
peak to AHP duration (microseconds)
d
% of NS and BS Units
% NS Units
% BS Units
[%] NS and BS
18.52
23.18
18.28
All SU
Stimulus SU
CRP SU
Unit Selection Criterion
e
All CRP Units
% of NS and BS Units
NS
BS
Average Waveforms - NS and BS Units
waveform amplitude
time
number of units
peak to AHP duration (microseconds)
f
% of NS and BS Units
% NS Units
% BS Units
[%] NS and BS
17.42
16.48
24.32
17.68
17.59
CRP -1
CRP -2
CRP -3
CRP -4
CRP -5
Unit Selection Criterion

Supplementary Figure 10: **Proportion of narrow spiking (NS) and broad spiking (BS) units among functionally different neuronal populations.** For every single unit (N = 1285), the waveforms recorded on the four different channels of a tetrode were extracted. The spike width was calculated as the temporal distance between the peak of the action potential and the trough of the hyperpolarization[2] and the waveform with longest spike width corresponding to every single unit was selected for further analysis. (a) A histogram of the spike widths (of this largest waveform) displayed a typical bimodal distribution corresponding to the NS and BS units. A k-means based clustering approach (after removing the single units in the outlier bin (N = 34) with the longest spike widths) with a 1000 replicates was utilized for identifying the two clusters corresponding to NS putative interneurons (red) and BS putative pyramidal cells (black). The proportion of NS and BS units are displayed as a pie plot (upper left inset), while the average waveforms corresponding to the two unit subtypes are plotted as an inset figure on the upper right. (b) Displayed are example waveforms of two NS units and BS units in red and black respectively. (c) and (e) display the same features as in (a) for stimulus selective neuronal population as well as the task-phase related (CRP) units. (d) This analysis revealed a similarly low proportion of NS units as compared to BS cells among different neuronal populations. (f) The proportion of NS units was lower than BS units and these proportions were remarkably similar among the neuronal populations clustered to the different CRPs.

SUPPLEMENTARY DISCUSSION

NNMF as a method for clustering neuronal response patterns

Although the method of NNMF was initially developed to extract certain characteristic parts from pictures of faces[3], its application to neuroscientific data has been growing. It has been recently used as a cell detection algorithm on calcium imaging data[4], to analyze network properties of a cortical region by embedding spike trains into an NNMF space[5], and for investigation of synchronized bursts occurring among dissociated cortical neurons in vitro[6]. However, it is to the best of our knowledge; the first time that the NNMF based classification approach[3, 7, 8] has been utilized for analyzing and classifying patterns of neuronal PSTHs. A key advantage of this methodology with respect to another kind of linear decomposition such as PCA is the interpretability of the resulting components as 'typical' neuronal responses. This interpretability is ensured by enforcing non-negativity of the components, thus preserving a key property of the original unit responses. The validity of this approach is further justified in this application by the observation that most units had their activity focused on a relatively small part of the trial duration, such that only a relatively small proportion of the units are active for a given task phase. This sparse activity of LPFC in task phase encoding may be related to previous results on sparse coding in sensory areas[9] and is also a feature of the data that helps NNMF algorithms to converge to a unique solution[10, 11]. In addition, such a methodology permits the isolation of strikingly different response patterns present in the data, thus allowing us to automatically extract generic response properties, which may not be obvious upon visual scrutiny of the averaged population activity. Further, as the electrophysiological methods advance and become viable for recording the electrical activity simultaneously from several hundred neurons or sites with multi electrode arrays[12, 13], automated algorithms for visualization, classification and presentation of neuronal

responses are increasingly needed[14, 15]. The computational approach presented here could therefore be utilized for offline and potentially for online classification and visualization of large scale electrophysiological data.

Sequential activity patterns in the various regions of the brain

Interestingly, similar sequential patterns of activity have been observed in various regions of the brain. These include the hippocampus[16, 17] of the rodent and in the temporal lobe of the macaque[18] attributed to the flow of time, in the olfactory system involved in encoding identity of odor stimuli[19, 20, 21], rodent gustatory cortex upon presentation of natural taste stimuli[22], during decision making [23] and object construction task[24] in the parietal cortex, and in the medial prefrontal cortex during memory guided saccade paradigm[25]. Although such sequential patterns of activity are observed in many different regions of the brain in diverse neural processes, it remains to be seen, if the structure of neuronal discharge fluctuations also displays similarity across different paradigms and various areas in the brain. If such a ubiquitous network encoding principle exists, it could provide a common underlying mechanism for the sequential responses found so pervasive among neuronal activity in the brain.

SUPPLEMENTARY REFERENCES

1. Chelaru MI, Dragoi V. Negative Correlations in Visual Cortical Networks. *Cerebral cortex* **26**, 246-256 (2016).

2. Hussar CR, Pasternak T. Flexibility of sensory representations in prefrontal cortex depends on cell type. *Neuron* **64**, 730-743 (2009).

3. Lee DD, Seung HS. Learning the parts of objects by non-negative matrix factorization. *Nature* **401**, 788-791 (1999).

4. Maruyama R, *et al*. Detecting cells using non-negative matrix factorization on calcium imaging data. *Neural Networks* **55**, 11-19 (2014).

5. Wei J, Bai W, Liu T, Tian X. Functional connectivity changes during a working memory task in rat via NMF analysis. *Front Behav Neurosci* **9**, 2 (2015).

6. Yada Y, Kanzaki R, Takahashi H. State-Dependent Propagation of Neuronal Sub-Population in Spontaneous Synchronized Bursts. *Front Syst Neurosci* **10**, 28 (2016).

7. Paatero P, Tapper U. Positive Matrix Factorization - a Nonnegative Factor Model with Optimal Utilization of Error-Estimates of Data Values. *Environmetrics* **5**, 111-126 (1994).

8. Dhillon IS, Sra S. Generalized nonnegative matrix approximations with Bregman divergences. In: *Proceedings of the 18th International Conference on Neural Information Processing Systems*, MIT Press (2005).

9. Olshausen BA, Field DJ. Sparse coding with an overcomplete basis set: a strategy employed by V1? *Vision research* **37**, 3311-3325 (1997).

10. Donoho DS, C. When does non-negative matrix factorization give a correct decomposition into parts? In: *Advances in neural information processing systems 16: proceedings of the 2003 conference*, MIT Press (2004).

11. Theis FJ, Stadlthanner K, Tanaka T. First results on uniqueness of sparse non-negative matrix factorization. In: *Signal Processing Conference, 2005 13th European* (2005).

12. Pine J. A History of MEA Development, Advances in Network Electrophysiology. Springer US (2006).

13. Miller EK, Wilson MA. All My Circuits: Using Multiple Electrodes to Understand Functioning Neural Networks. *Neuron* **60**, 483-488 (2008).

14. Armananzas R, Ascoli GA. Towards the automatic classification of neurons. *Trends Neurosci* **38**, 307-318 (2015).

15. Kobak D, *et al.* Demixed principal component analysis of neural population data. *Elife* **5**, (2016).

16. Eichenbaum H. Time cells in the hippocampus: a new dimension for mapping memories. *Nature reviews Neuroscience* **15**, 732-744 (2014).

17. Pastalkova E, Itskov V, Amarasingham A, Buzsaki G. Internally generated cell assembly sequences in the rat hippocampus. *Science* **321**, 1322-1327 (2008).

18. Sakon JJ, Naya Y, Wirth S, Suzuki WA. Context-dependent incremental timing cells in the primate hippocampus. *Proceedings of the National Academy of Sciences of the United States of America* **111**, 18351-18356 (2014).

19. Laurent G, Wehr M, Davidowitz H. Temporal representations of odors in an olfactory network. *The Journal of neuroscience : the official journal of the Society for Neuroscience* **16**, 3837-3847 (1996).

20. Wehr M, Laurent G. Odour encoding by temporal sequences of firing in oscillating neural assemblies. *Nature* **384**, 162-166 (1996).

21. Laurent G. Olfactory network dynamics and the coding of multidimensional signals. *Nature reviews Neuroscience* **3**, 884-895 (2002).

22. Jones LM, Fontanini A, Sadacca BF, Miller P, Katz DB. Natural stimuli evoke dynamic sequences of states in sensory cortical ensembles. *Proceedings of the National Academy of Sciences of the United States of America* **104**, 18772-18777 (2007).

23. Harvey CD, Coen P, Tank DW. Choice-specific sequences in parietal cortex during a virtual-navigation decision task. *Nature* **484**, 62-68 (2012).

24. Crowe DA, Averbeck BB, Chafee MV. Rapid sequences of population activity patterns dynamically encode task-critical spatial information in parietal cortex. *The Journal of neuroscience : the official journal of the Society for Neuroscience* **30**, 11640-11653 (2010).

25. Campos M, Breznen B, Andersen RA. A neural representation of sequential states within an instructed task. *Journal of neurophysiology* **104**, 2831-2849 (2010).
